# Four SNPs in the CHRNA3/5 Alpha-Neuronal Nicotinic Acetylcholine Receptor Subunit Locus Are Associated with COPD Risk Based on Meta-Analyses

**DOI:** 10.1371/journal.pone.0102324

**Published:** 2014-07-22

**Authors:** Kai Cui, Xiaoyan Ge, Honglin Ma

**Affiliations:** School of Public Health, Liaoning Medical University, Jinzhou, Liaoning, P.R. China; The Ohio State University, United States of America

## Abstract

**Background:**

Several single nucleotide polymorphisms (SNPs) in an α-neuronal nicotinic acetylcholine receptor subunit (CHRNA3/5) were identified to be associated with chronic obstructive pulmonary disease (COPD) in a study based on a Norwegian population. However, results from subsequent studies have been controversial, particularly in studies recruiting Asians. In the present study, we conducted a comprehensive search and meta-analyses to identify susceptibility SNPs for COPD in the CHRNA3/5 locus.

**Methods:**

A comprehensive literature search was conducted to find studies that have reported an association between SNPs in the CHRNA3/5 locus and COPD risk. Pooled odds ratios (ORs) with 95% confidence intervals (CIs) for each SNP were calculated with the major allele or genotype as the reference group. The influence of individual studies on pooled measures was assessed, in addition to publication bias.

**Results:**

A total of 12 articles with 14 eligible studies were included in this analysis. Association between 4 SNPs in the CHRNA3/5 locus and COPD was evaluated and included rs1051730, rs8034191, rs6495309, and rs16969968. Significant associations between the 4 SNPs and COPD were identified under allele (rs1051730: OR = 1.14, 95%CI = 1.10–1.18; rs8034191: OR = 1.29, 95%CI = 1.18–1.41; rs6495309: OR = 1.26, 95%CI = 1.09–1.45; rs16969968: OR = 1.27, 95%CI = 1.17–1.39) and genotype models. Subgroup analysis conducted for rs1051730 showed a significant association between this SNP and COPD risk in non-Asians (OR = 1.14, 95%CI = 1.10–1.18), but not Asians (OR = 1.23, 95%CI = 0.91–1.67). Rs1051730 and rs6495309 were also significantly associated with COPD after adjusting for multiple variables, including age and smoking status.

**Conclusion:**

Our results indicate that 4 SNPs in the CHRNA3/5 locus are associated with COPD risk. Rs1051730 was particularly associated with COPD in non-Asians, but its role in Asians still needs to be verified. Additional studies will be necessary to assess the effect of rs6495309 on COPD. Although rs1051730 and rs6495309 were shown to be independent risk factors for COPD, validation studies should be performed.

## Introduction

Tobacco smoking is a major risk factor for development of chronic obstructive pulmonary disease (COPD) [1]. Nicotine is the major reinforcing component of tobacco smoking and acts through neuronal nicotinic acetylcholine receptors (nAChRs) [2]. Studies have shown that genetic variants in the α-nAChR 3/5 subunit (CHRNA3/5) locus can influence nicotine dependence, smoking behavior, and lung cancer [3]–[6]. Pillai et al identified 2 susceptibility single nucleotide polymorphisms (SNPs) in the CHRNA3/5 locus, rs1051730 and rs8034191, as significant locations that may influence COPD in a recent genome-wide association (GWA) study [7]. Several subsequent studies have assessed the association between polymorphisms in this locus and COPD risk in different ethnicities and identified several other SNPs in the CHRNA3/5 locus as being associated with COPD risk. However, several studies have yielded contradictory results, particularly regarding Asian ethnic populations, possibly due to limited sample sizes and variations in study design. While most association studies recruit Caucasians, several SNPs, such as rs1051730 and rs8034191, are extremely rare in Asians [8]–[10]. Thus, meta-analyses are necessary to quantitatively synthesize the results. In the present study, we conducted a comprehensive search and meta-analyses to confirm the effect of CHRNA3/5 variations on COPD risk.

## Materials and Methods

### Search strategy

A comprehensive search was performed in several databases, including Pubmed, ISI Web of Science, CNKI (China National Knowledge Infrastructure), CBM (China Biology Medical Literature database), and Wangfang Data for potentially relevant studies published in English or Chinese. An upper date limit of Dec 1, 2013 was applied, and a lower date limit was not specified. A combination of the following Medical Subject Headings (MeSH) terms and key words were used: “pulmonary disease, chronic obstructive”, “lung disease, obstructive”, “COPD”, “chronic obstructive pulmonary disease”, “receptors, nicotinic”, “nicotinic receptor subunit”, “nAChR”, “CHRNA”, and “15q25”. Bibliographies of relevant articles were manually reviewed to identify additional studies that were not captured in the key word search.

### Study eligibility

Studies that assessed the association between any SNP in the CHRNA3/5 locus and COPD risk and reported genotype count, allele frequency, or odds ratios (ORs) with 95% confidence intervals (CIs) were included in this study. Case-control, cohort, or population-based studies were eligible, although studies without a control group or based on family or sibling pairs were excluded. The study with the largest sample size was included when 2 or more studies contained the same data set. All potentially available articles were independently identified by 2 reviewers to determine study eligibility. Consensus with a third reviewer was used to resolve disagreements regarding eligibility.

### Data extraction and quality assessment

Extracted data included the following: name of first author, publication year, country in which the study was conducted, ethnicity of the study population, type of study design, source of control subjects, mean age, male percentage, smoking status, genotyping methods, definition of COPD, matched methods, total number of cases and controls, genotype count, allele frequency, and ORs with 95%CIs adjusted for multiple variables.

The Newcastle-Ottawa quality assessment scale was used to evaluate the quality of each study. Assessment for case-control studies was based on 3 major components, including selection of cases and controls, comparability of cases and controls, and exposure. Prospective cohort studies were evaluated on another scale consisting of 3 components, including selection of exposed and non-exposed populations, comparability of cohorts, and outcome. The assessment was conducted independently by 2 reviewers, and disagreements were resolved by consensus with a third reviewer.

### Statistical analysis

SNPs reported by more than 3 independent studies underwent further meta-analysis. Fisher's exact test was used to analyze deviations from Hardy-Weinberg equilibrium (HWE) for each genotype distribution in the control group. The inverse variance-weighted mean was used to calculate pooled ORs with 95%CIs for each SNP to represent association with risk of COPD under allele, homozygote, and heterozygote genotype models. The major allele or genotype was used as the reference group. The I^2^ statistic was used to evaluate heterogeneity among studies, which describes the proportion of total variation attributable to between-study heterogeneity. I^2^>50% was considered significant statistical heterogeneity, and pooled ORs were calculated using the DerSimonian and Laird random effect model. For I^2^<50%, the fixed effect model was used. If needed, subgroup analyses were conducted to explore the source of heterogeneity. One-way sensitivity analyses in which a single study was omitted were employed to assess robustness of the pooled estimators in response to removal of an individual study. When the point estimate of the omitted analysis was outside the 95%CI of the pooled measures, the individual study was suspected of excessive influence. Begg's test was conducted to estimate publication bias and displayed as a funnel plot. All statistical analyses were performed with STATA version 9.2 (Stata Corporation, College Station, TX, USA). All reported probabilities were two-tailed, with P<0.05 considered statistically significant.

## Results

### Characteristics of included studies

**Figure 1:**
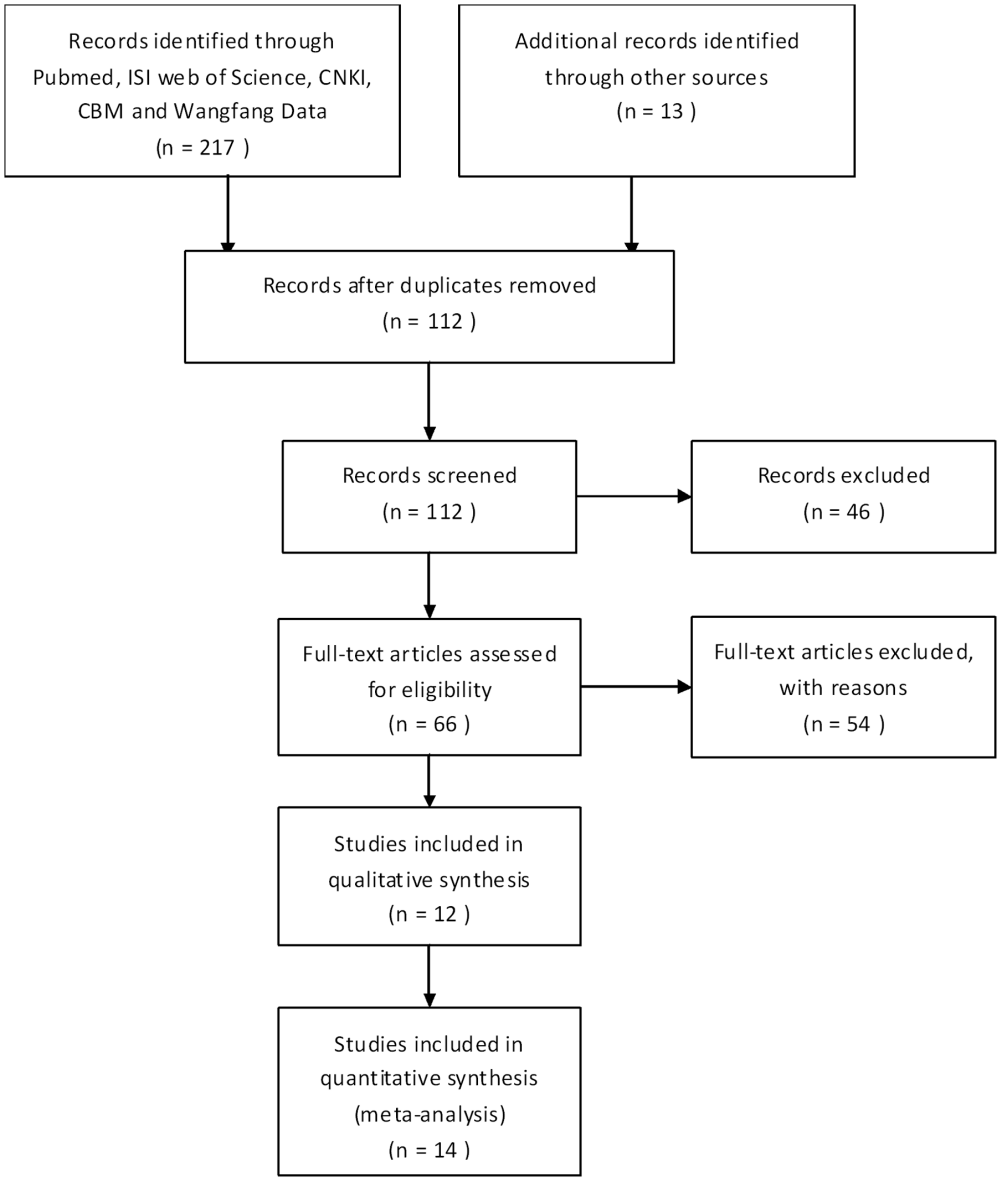
Study identification flow diagram.

A study identification flow diagram is shown in Figure 1. A total of 12 articles [7], [9], [11]–[20] consisting of 14 eligible studies satisfied the inclusion criteria. Only 1 article was published in Chinese [14]. Four SNPs were studied, including 9 studies on rs1051730 [11]–[13], [15]–[18], [20], 5 on rs8034191 [7], [13], [18], [20], 4 on rs6495309 [9], [14], [17], and 3 on rs16969968 [11], [18]–[19]. A total of 6 studies assessed associations between SNPs in the CHRNA3/5 locus and COPD risk in Asian populations [9], [14]–[15], [17]–[18]. A total of 10 studies were case-control, while all others were population-based. Most of the studies used the Global Initiative for Chronic Obstructive Lung Disease (GOLD) or American Thoracic Society (ATS) to diagnose or define COPD patients. General characteristics, quality assessments for each study, and allele and genotype distributions of each SNP are shown in Tables S1 and S2.

### Quantitative synthesis

**Figure 2:**
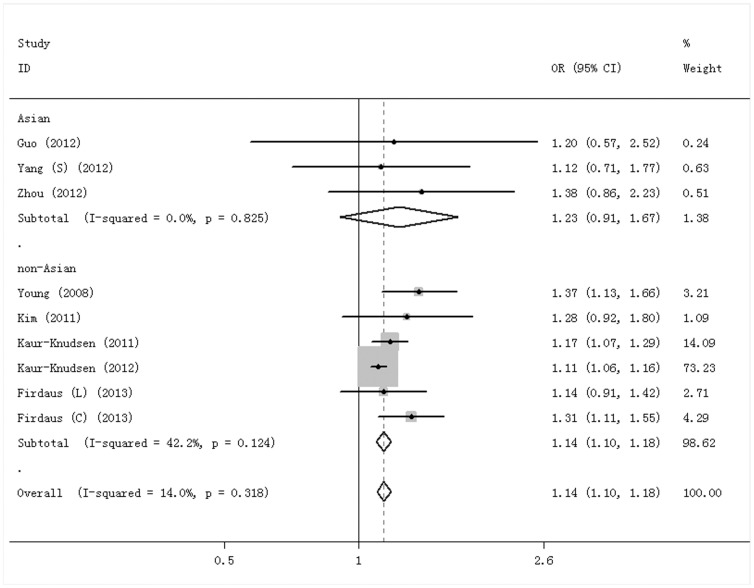
Forest plot for rs1051730 T vs C. The fixed effect model was used in both overall and subgroup analyses. Each box represents the OR point estimate, and its area is proportional to the weight of the study. Diamond and broken lines represent the overall estimate, with 95%CI represented by the width. The solid vertical line is set at the null value (OR = 1.0).

As shown in Table S2, none of the studies deviated from HWE for each genotype distribution in the control group. For rs1051730, 9 studies were included, and the pooled OR with 95%CI was calculated with the C allele as the reference group. The I^2^ was 14.0%, and a fixed effect model was used. Allele distribution was significantly different between the groups, with an OR of 1.14 for the T vs C allele (95%CI = 1.10–1.18, P = 0.000) (Figure 2). Considering the genetic diversity between different ethnic populations, subgroup analysis was employed according to the ethnicity of the study population (Asian and non-Asian). In non-Asians, the T allele was associated with a significantly increased risk of COPD (OR = 1.14, 95%CI = 1.10–1.18, P = 0.000). However, a statistically significant difference was not observed in Asians (OR = 1.23, 95%CI = 0.91–1.67, P = 0.18).

**Figure 3:**
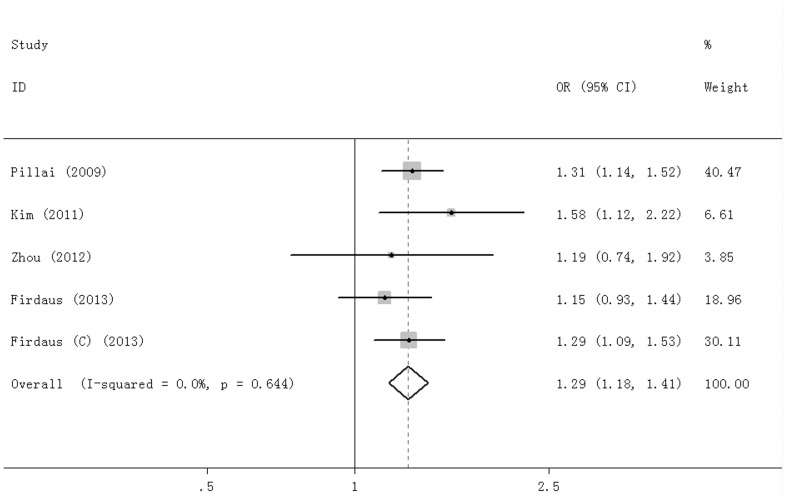
Forest plot for rs8034191 C vs T allele.

For rs8034191, 5 studies were included. The I^2^ was 0.0%, and the fixed effect model was used to calculate association of the C allele with COPD risk compared with the T allele. The C allele was significantly associated with the risk of COPD (OR = 1.29, 95%CI = 1.18–1.41, P = 0.000) (Figure 3).

**Figure 4:**
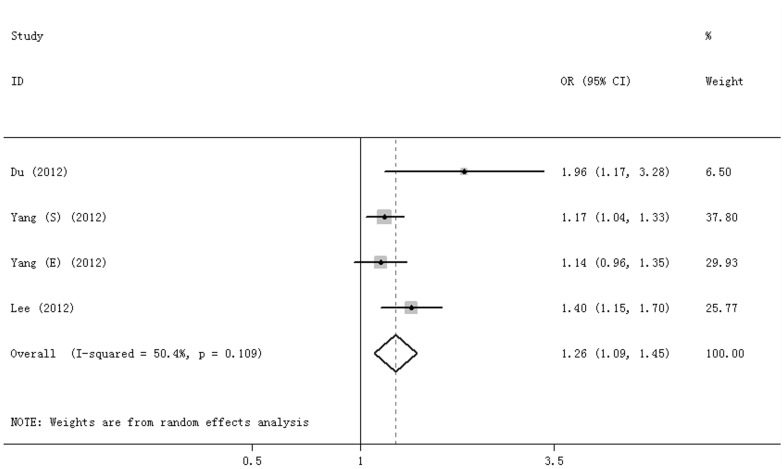
Forest plot for rs6495309 C vs T allele.

For rs6495309, 4 studies were included, and all were based on an Asian population. Marginal heterogeneity was observed (I^2^ = 50.4%), and a random effect model was used to calculate association of the C allele with COPD risk compared with the T allele. The C allele was associated with a significantly increased risk of COPD (OR = 1.26, 95%CI = 1.09–1.45, P = 0.001) (Figure 4).

**Figure 5:**
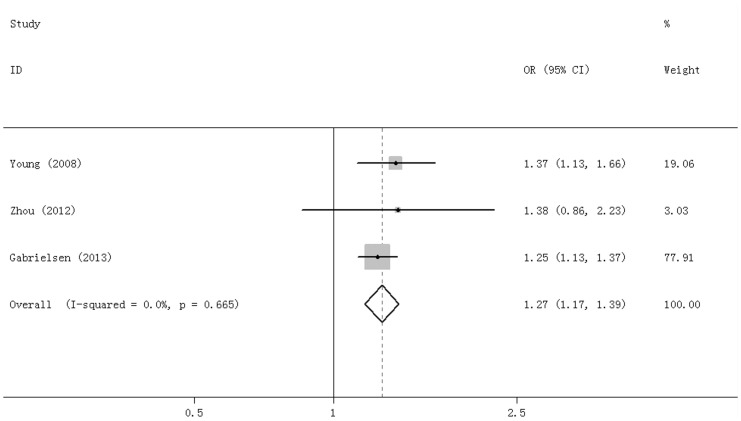
Forest plot for rs16969968 C vs T allele.

For rs16969968, 3 studies were included. The I^2^ was 0.0%, and the OR for the A allele regarding risk of COPD compared with the G allele was 1.27 (95%CI = 1.17–1.39, P = 0.000) (Figure 5).

ORs with 95%CIs for heterozygote and homozygote genotypes were also calculated (Table 1). Under both heterozygote or homozygote genotype models of each SNP, results were similar to those of the allele models, except for the rs6495309 heterozygote genotype. The CT genotype increased the risk of COPD, but the difference was not significant (OR = 1.12, 95%CI = 0.85–1.49, P = 0.424).

**Table 1:** ORs with 95%CIs for heterozygote and homozygote genotypes of each SNP.

SNPs	Inheritance model	n of eligible studies	I^2^ (%)	OR with 95%CI	P
rs1051730	Homozygote model: TT/CC	7	0.0	1.30 (1.20–1.41)	0.000
	Heterozygote model: CT/CC	9	42.2	1.13 (1.07–1.19)	0.000
rs8034191	Homozygote model: CC/TT	4	0.0	1.56 (1.27–1.92)	0.000
	Heterozygote model: CT/CC	5	21.9	1.37 (1.20–1.56)	0.000
rs6495309	Homozygote model: CC/TT	4	56.9	1.53 (1.14–2.06)	0.005
	Heterozygote model: CT/TT	4	59.6	1.12 (0.85–1.49)	0.424
rs16969968	Homozygote model: AA/GG	2	0.0	1.56 (1.30–1.88)	0.000
	Heterozygote model: AG/GG	3	0.0	1.32 (1.17–1.49)	0.000

To adjust for other confounding factors, such as smoking, age, and gender, we conducted meta-analyses for eligible studies that reported ORs with 95%CIs adjusted for multiple variables. For rs1051730 and rs6495309, 3 studies were included in the meta-analyses. Pooled ORs with 95%CIs were 1.11 (1.01–1.20) and 1.31 (1.08–1.54), respectively (Table 2).

**Table 2:** Pooled ORs with 95%CIs for multiple variable adjustment.

SNPs	Author (year)	Adjusted variables	OR with 95%CI adjusted for multiple variables	Pooled OR with 95%CI
rs1051730 a	Yang (S) (2012)	age, gender, smoking status, drinking status	1.10 (0.67–1.84)	1.11 (1.01–1.20)
	Zhou (2012)	age, gender, BMI, smoking status, pack-years of smoking	1.40 (0.86–2.30)	
	Kaur-Knudsen (2012)	age, gender, cumulative tobacco consumption	1.1 (1.0–1.2)	
rs6495309 ^b^	Yang (S) (2012)	age, gender, smoking status, drinking status	1.27 (1.01–1.61)	1.31 (1.08–1.54)
	Yang (E) (2012)	age, gender, smoking status, drinking status	1.23 (0.88–1.70)	
	Lee (2012)	age, pack-years of smoking	2.00 (1.32–3.03)	

a : Heterozygote inheritance model (CT vs CC genotype); ^b^: Homozygote inheritance model (CC vs TT genotype).

### Sensitivity analyses and publication bias

**Figure 6:**
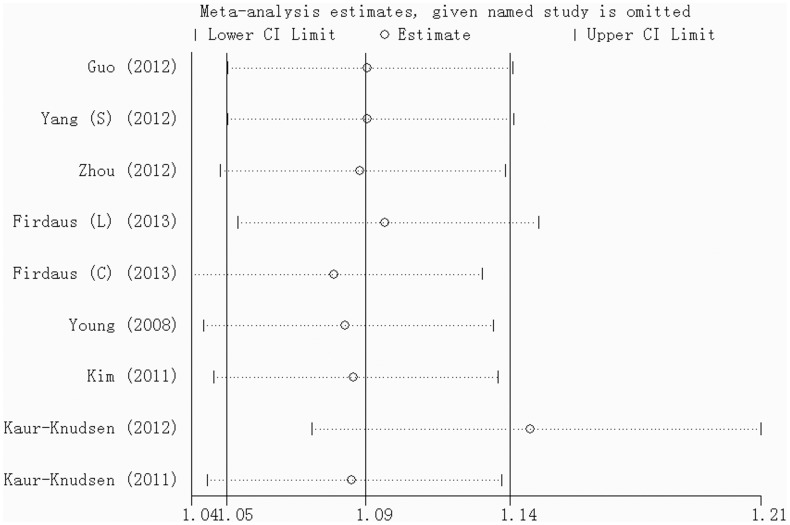
Sensitivity analysis for the rs1051730 heterozygote genotype (CT vs CC).

No strong influence of any single study was detected after it was omitted, except for the rs1051730 heterozygote genotype (Figure 6). After the 2012 study by Kaur-Knudsen et al was omitted, the pooled OR was outside the 95%CI of the pooled measures, suggesting that this study had an excessive influence on the results. The pooled OR increased after omission of this individual study (OR = 1.24, 95%CI = 1.13–1.38, P = 0.000). Considering the small number of studies included in the meta-analyses, Begg's test was conducted to assess potential publication bias when more than 4 studies were included. No obvious evidence of publication bias was detected for any of the allele, homozygote, and heterozygote models in each SNP, except for rs6495309 (Table 3).

**Table 3:** Publication bias in meta-analyses for each inheritance model.

SNP	Inheritance model	n of eligible studies	P for Begg's test
rs1051730	Allele model: T vs C allele	9	0.835
	Homozygote genotype: TT vs CC	7	0.881
	Heterozygote genotype: CT vs CC	9	0.835
rs8034191	Allele model: C vs T allele	5	0.624
	Homozygote genotype: CC vs TT	4	1.000
	Heterozygote genotype: CT vs TT	5	1.000
rs6495309	Allele model: C vs T allele	4	0.042
	Homozygote genotype: CC vs TT	4	0.042
	Heterozygote genotype: CT vs TT	4	0.174

## Discussion

In 2011, Zhang et al conducted a meta-analysis to assess the effect of rs1051730 in the CHRNA3/5 locus on COPD [21] based on data from published studies and 2 GWA studies (Bergen and COPCETI cohort [7], [22]). The results indicated that rs1051730 was a susceptibility variant for COPD. However, studies included in the Zhang et al meta-analyses were all based on Caucasians. Several subsequent association studies recruiting Asians have been conducted, but results have been controversial and inconclusive. It may be because polymorphism frequencies, such as rs1051073 and rs8034191, are lower in Asians than in Caucasians [8]–[10]. This implies differences in genetic susceptibility between the 2 ethnic populations and suggests that there might be other variants in the CHRNA3/5 locus that could influence COPD. In this study, we conducted a comprehensive search for studies about genetic variants in the CHRNA3/5 locus based on both Caucasian and Asian populations to assess the association of these polymorphisms with COPD risk. COPD likely results from a cumulative effect of environmental and genetic factors and gene-by-environment interactions [23]. The subjects in recent studies are former or current smokers, and as such it is difficult to identify whether variants in the CHRNA3/5 locus have a direct influence on COPD independent of smoking behavior or nicotine dependence.

While searching for eligible studies, we found 17 SNPs in the CHRNA3/5 locus that were reported to be associated with COPD, including rs1051730, rs8034191, rs6495309, rs16969968, rs578776, rs12910984, rs660652, rs1317286, rs569207, rs12914008, rs931794, rs938682, rs17486278, rs7180002, rs951266, rs684513, and rs588765. However, only the former 4 SNPs were studied in at least 5 individual studies. For each SNP included in this study, we compared allele and genotype distribution between cases and controls. Our results showed that the T allele in rs1051730 was associated with COPD risk, which was similar for both homozygote and heterozygote genotypes. This conclusion was consistent with the studies by Pillai et al and Zhang et al. In the present study, we found the polymorphism frequency was far lower in Asians. The minor allele frequency (MAF) in the non-Asian subgroup was greater than 0.48. The largest MAF was 0.031 from Guo's study in an Asian subgroup. We further evaluated the association between rs1051730 and COPD in different ethnic subgroups and showed that the T allele increased COPD risk, particularly in non-Asians. However, based on the results, rs1051730 does not appear to be associated with COPD in Asians. Because only 3 of the studies included an Asian subgroup and the MAF was lower than in non-Asians, additional studies are still needed to confirm the association between this polymorphism and COPD risk in Asians.

Rs8034191 is another susceptibility SNP identified in the study of Pillai et al. Our results showed that the rs8034191 C allele was associated with COPD risk under any of the inheritance models, including allele, homozygote, and heterozygote genotypes. Given that there was only one study based on an Asian population, subgroup analysis for different ethnicities was not conducted. Similar to rs1051730, the MAF of this SNP was 0.031 from Zhou's study based on Chinese subjects, while the lowest MAF in the studies with non-Asian participants was 0.51 (study of Kim et al), which was based on Caucasians. We suspect that there is a stronger association between rs8034191 and COPD risk in non-Asians than Asians, which should be confirmed through additional studies.

Based on the results of rs6495309 and rs16969968, an association between SNPs and COPD risk was detected. The rs6495309 C allele and rs16969968 A allele were considered risk alleles that increased the risk of COPD. The studies included in meta-analyses for rs6495309 were mainly based on Chinese or Korean populations, and none of the studies reported an association between rs6495309 and COPD in Caucasians or non-Asians, including the GWA studies, indicating that this SNP is likely an important susceptibility variant for COPD in Asians.

We tried to detect whether these SNPs in the CHRNA3/5 locus were associated with COPD risk by direct or indirect influence. We pooled the ORs with 95%CIs adjusted for multiple variables, including smoking status and pack-years of smoking. Eligible data were found in the rs1051730 heterozygote genotype and rs6495309 homozygote genotype. Although the number of included studies was small, the results still suggest that these variants are associated with COPD risk, independent of smoking behavior or nicotine dependence. This conclusion needs to be further confirmed with additional studies based on never smokers.

In sensitivity analyses, the study by Kaur-Knudsen et al had a strong influence on pooled measures under an inheritance model of rs1051730, with the pooled OR increasing after omitting this individual study. However, the pooled OR was still statistically significant at P = 0.05 and the results after omitting the study still confirmed an association between the polymorphism and COPD. Publication bias was detected under some inheritance models of rs6495309. In this meta-analysis, only 4 studies on the association of rs6495309 with COPD were included, and the bias could be caused by the small number of studies. According to our results, rs6495309 is associated with COPD, but future studies will need to confirm this conclusion regarding publication bias.

Based on our study, 4 SNPs in the CHRNA3/5 locus were associated with COPD risk. Among the 4 SNPs, the rs1051730 T allele was considered a risk allele, particularly in non-Asians. However, the effect of both rs1051730 in Asians and rs6495309 on COPD risk needs to be confirmed by further studies. Although rs1051730 and rs6495309 were found to be independent risk factors for COPD, this effect will need to be confirmed.

## Supporting Information

Table S1 **General characteristics and quality assessment of each study.**
(DOC)

Table S2 **Distribution of the SNP alleles and genotypes.**
(DOC)

Checklist S1 **PRISMA meta-analysis checklist.**
(DOCX)
