# Supplementary material for: Four SNPs in the CHRNA3/5 Alpha-Neuronal Nicotinic Acetylcholine Receptor Subunit Locus Are Associated with COPD Risk Based on Meta-Analyses
Source: PLoS One. 2014 Jul 22;9(7):e102324. doi: 10.1371/journal.pone.0102324 (PMC4106784; doi:10.1371/journal.pone.0102324)
Supplement: Table S1 — General characteristics and quality assessment of each study. (DOC) [file pone.0102324.s001.doc]

Table S1. General characteristics and quality assessment of each study.

| First author (Publication year) | Country of study population | Name of study | Ethnicity of population | Study type | COPD definition | Smoking history | Match methods of study | Genotyping methods | Quality  score |
| --- | --- | --- | --- | --- | --- | --- | --- | --- | --- |
| Young (2008) | New Zealand | - | Caucasian | case-control | post-FEV1<80%predicted, FEV1/FVC<70% | ≥15 py | NS | Sequenom’s iPLEX with the MassARRAY | 6 |
| Pillai (2009) | Norway | - | NS | case-control | post-FEV1<80%predicted, FEV1/FVC<70% | ≥2.5 py | NS | Illumina’s HumanHap550 genotyping BeadChip | 6 |
| Kim (2011) | USA | COPDGene | non-Hispanic White and African | Population-based | post-FEV1<80%predicted, FEV1/FVC<70% | ≥ 10 py | NS | TaqMan genotyping assay | 7 |
| Kaur-Knudsen (2011) | Denmark | Copenhagen City Heart Study | NS | Population-based | ICD-8491 to 492 and ICD-10J41 to J44 | NS | NS | TaqMan genotyping assay | 9 |
| Guo (2012) | China | - | Asian | case-control | FEV1/FVC≤70% | ≥20 py | NS | Multiplex PCR-Mass-Array™ Technology | 7 |
| Du (2012) | China | - | Asian | case-control | FEV1<80%predicted FEV1/FVC<70% | Current smoker | Gender, age | PCR-RFLP | 6 |
| Yang (2012) | China (S) ^a^ | - | Asian | case-control | FEV1/FVC≤70% | NS | Gender, age | PCR-RFLP | 7 |
|  | China (E) ^b^ | - | Asian | case-control | FEV1/FVC≤70% | NS | Gender, age | PCR-RFLP | 6 |

Table S1. General characteristics and quality assessment for each study included in this study (continued).

| Zhou (2012) | China | - | Asian | case-control | FEV1<80%predicated FEV1/FVC<70% | NS | Gender, age, smoking status | Sequenom’s iPLEX with the Mass ARRAY spectrometer | 5 |
| --- | --- | --- | --- | --- | --- | --- | --- | --- | --- |
| Lee (2012) | Korea | - | Asian | case-control | FEV1<80%predicted, FEV1/FVC<70% | Current or former smoker | NS | melting curve analysis using fluorescence labeled hybridization probes | 5 |
| Kaur-Knudsen (2012) | Denmark | Copenhagen General Population Study | NS | Population-based | FEV1/FVC<70% | Ever smoker | NS | TaqMan genotyping assay | 8 |
| Firdaus (2013) | Belgian | LEUVEN | NS | case-control | post-FEV1/FVC<70% | NS | Age, smoking status | iPLEX technology on a MassARRAY | 8 |
|  | Netherlands | COPACETI cohort | NS | Population-based | post-FEV1/FVC<70% | NS | NS | Illumina's Human 610-Quad BeadChip | 6 |
| Gabrielsen (2013) | Norway | HUNT |  | Population-based | pre-FEV1/FVC<70%, FEV1<80%predicted | NS | NS | TaqMan genotyping assay | 6 |

^a^: Study was based on population from the south of China; ^b^: Study was based on population from the east of China; post-FEV1: Post-bronchodilator forced expiratory volume at one second; pre-FEV1: Pre- bronchodilator forced expiratory volume at one second; FVC: Forced vital capacity; py: Pack-years; ICD: WHO International Classification of Diseases; RFLP: Restriction fragment length polymorphism; NS: no statement.
