# Supplementary material for: Four SNPs in the CHRNA3/5 Alpha-Neuronal Nicotinic Acetylcholine Receptor Subunit Locus Are Associated with COPD Risk Based on Meta-Analyses
Source: PLoS One. 2014 Jul 22;9(7):e102324. doi: 10.1371/journal.pone.0102324 (PMC4106784; doi:10.1371/journal.pone.0102324)
Supplement: Table S2 — Distribution of the SNP alleles and genotypes. (DOC) [file pone.0102324.s002.doc]

Table S2. Distribution of the SNP alleles and genotypes.

| SNP | Author (year) | Age Case/Control | Male % Case/Control | Smoking status (pack-years) Case/Control | Sample Size  Case/Control | Genotype distribution  (11/12/22^e^) | | Allele frequencies (1/2^e^) | | P for  HWE |
| --- | --- | --- | --- | --- | --- | --- | --- | --- | --- | --- |
|  |  |  |  |  |  | Case | Control | Case | Control |  |
| rs1051730 ^f^ | Young (2008) | 66±9/65±10 | 59/60 | 47±20/40±19 | 445/475 | 166/219/60 | 225/205/45 | 551/339 | 655/295 | 0.86 |
|  | Kim (2011) | 59±8/54±7 | 55/54 | 52±29/37±19 | 138/164 | 50/72/16 | 79/65/20 | 172/104 | 223/105 | 0.25 |
|  | Kaur-Knudsen (2011) | NS | NS | NS | 1034/9037 | 421/472/141 | 4071/3995/971 | 1314/754 | 12137/5937 | 0.84 |
|  | Guo (2012) | 61±10/58±12 | 98/99 | 41±34/38±17 | 331/207 | 311/19/1 | 196/11/0 | 641/21 | 403/11 | 0.69 |
|  | Yang (S) ^a^ | NS | 60/60 | NS | 1027/1061 | 988/39/0 | 1025/36/0 | 2015/39 | 2086/36 | 0.57 |
|  | Zhou (2012) | 63±9/61±10 | 71/69 | NS | 488/687 | 454/34/0 | 652/35/0 | 942/34 | 1339/35 | 0.49 |
|  | Kaur-Knudsen (2012) | NS | NS | NS | 5818/26510 | 2512/2607/699 | 12165/11639/2706 | 7631/4005 | 35969/17051 | 0.31 |
|  | Firdaus (L) ^c^ (2013) | 65/62 | 71/78 | 48/46 | 645/229 | 244/296/105 | 93/106/30 | 784/506 | 292/166 | 0.98 |
|  | Firdaus (C) ^d^  (2013) | NS | NS | NS | 540/684 | 204/271/65 | 326/291/67 | 679/401 | 943/425 | 0.86 |
| rs8034191 ^g^ | Pillai (2009) | 65±10/55±10 | 60/50 | 32±19/19±13 | 823/810 | 326/375/122 | 391/328/91 | 1027/619 | 1110/510 | 0.08 |
|  | Kim (2011) | 59±8/54±7 | 55/54 | 52±29/37±19 | 144/155 | 49/78/17 | 77/67/11 | 176/112 | 221/89 | 0.49 |
|  | Zhou (2012) | 63±9/61±10 | 71/69 | NS | 488/687 | 456/32/0 | 649/38/0 | 944/32 | 1336/38 | 0.46 |
|  | Firdaus (L) (2013) | 65/62 | 71/78 | 48/46 | 653/230 | 238/300/115 | 90/109/31 | 776/530 | 289/171 | 0.82 |
|  | Firdaus (C) (2013) | NS | NS | NS | 544/683 | 205/274/65 | 326/286/71 | 684/404 | 938/428 | 0.48 |

Table S2. Distributions of the alleles and genotypes of SNPs in studies included in this study (continued).

| rs6495309 ^h^ | Du (2012) | 65±12/64±14 | 83/83 | NS | 60/60 | 20/28/12 | 10/28/22 | 68/52 | 48/72 | 0.83 |
| --- | --- | --- | --- | --- | --- | --- | --- | --- | --- | --- |
|  | Yang (S) ^a^ (2012) | NS | 60/60 | NS | 1025/1061 | 365/433/227 | 309/502/250 | 1163/887 | 1120/1002 | 0.10 |
|  | Yang (E) ^b^ (2012) | NS | 56/56 | NS | 486/616 | 168/204/114 | 176/292/148 | 540/432 | 644/588 | 0.21 |
|  | Lee (2012) | 64±8/62±8 | 100/100 | 44±22/32±16 | 406/394 | 140/198/68 | 105/189/100 | 478/334 | 399/389 | 0.42 |
| rs16969968 ^i^ | Young (2008) | 66±9/65±10 | 59/60 | 47±20/40±19 | 445/475 | 166/219/60 | 225/205/45 | 551/339 | 655/295 | 0.86 |
|  | Zhou (2012) | 63±9/61±10 | 71/69 | NS | 488/687 | 454/34/0 | 652/35/0 | 942/34 | 1339/35 | 0.49 |
|  | Gabrielsen (2013) | NS | NS | NS | 1063/5301 | 412/499/152 | 2420/2289/592 | 1323/803 | 7129/3473 | 0.15 |

^a^: Study was based on population from the south of China; ^b^: Study was based on population from the east of China; ^c^: Data were from LEUVEN study; ^d^: Data were from COPACETIC cohort study; ^e^: 1 for the major allele, 2 for the minor allele; HWE: Hardy-Weinberg equilibrium; NS: No statement; ^f^: Major vs minor allele (C vs T); ^g^: Major vs minor allele (T vs C); ^h^: Major vs minor allele (C vs T); ^i^: Major vs minor allele (G vs A).
